# *Talaromyces marneffei* infection with *IFNGR1* gene mutation in a patient with negative Anti-Interferon-γ autoantibodies^☆^

**DOI:** 10.1016/j.abd.2023.03.006

**Published:** 2023-11-03

**Authors:** Shiyang Li, Xianwei Cao, Zhuxiu Guo, Jian Wang, Jianbo Tong, Zhibin Zhang

**Affiliations:** Department of Dermatology, The First Affiliated Hospital of Nanchang University, Nanchang, Jiangxi, China

**Keywords:** HIV, Interferon-gamma, Mutation, Talaromyces

## Abstract

**Background:**

*Talaromyces Marneffei* (TM) is a rare opportunistic pathogen that mostly infects patients with low immunity compared to those with normal immunity. It may be related to immune deficiency or genetic factors.

**Objective:**

To evaluate the gene mutation of a patient infected with TM in an endemic area with negative anti-interferon-γ autoantibodies, and negative human immunodeficiency virus (HIV) infection.

**Methods:**

Extract deoxyribonucleic acid (DNA) samples from the patient's peripheral blood, detect the mutation gene by whole exome sequencing (WES), and carry out Sanger sequencing verification for the detected mutation gene.

**Results:**

The authors detected a mutation in the *IFNGR1* gene (NM_001363526.1) and validated the detected gene mutation using Sanger sequencing. The results showed a heterozygous mutation c.4C>T (p.L2F) located in the *IFNGR1* gene (NM_001363526.1).

**Study limitations:**

The mechanism of the *IFNGR1* gene has not been further investigated in this study.

**Conclusions:**

The *IFNGR1* gene mutation may be a potential risk factor for TM infection, and the presence of anti-interferon-γ autoantibodies can aggravate disease symptoms.

## Introduction

*Talaromyces Marneffei* (TM) is an opportunistic temperature-dependent biphasic pathogenic fungus that generally infects immunocompromised patients, predominantly human immunodefciency virus (HIV) infected patients.^1^ Among acquired immunodefciency syndrome (AIDS) patients, the infection rate is as high as 16.2% and is the leading cause of death.^2^ However, TM infection can also occur in HIV-negative individuals without significant immunosuppression. Host susceptibility to this infection may be due to immune deficiency or genetic factors. *IFNGR1* is a gene located on chromosome 6. The expression product of *IFNGR1* is an interferon-γ receptor, which combines with interferon-γ and participates in the activation of macrophages and anti-infection processes. The anti-interferon-γ autoantibodies block Interferon-γ (IFN-γ), resulting in compromised immunity or immune deficiency. This, in turn, makes the patient susceptible to pathogens.

In this study, the authors reported a rare case of endemic, non-HIV, non-immunosuppressive, anti-interferon-γ autoantibodies negative TM infection with *IFNGR1* gene mutation. The characteristics of TM infection cases with gene mutations or anti-interferon-γ autoantibodies in the literature were reviewed and summarized.

## Case report

**Figure 1:**
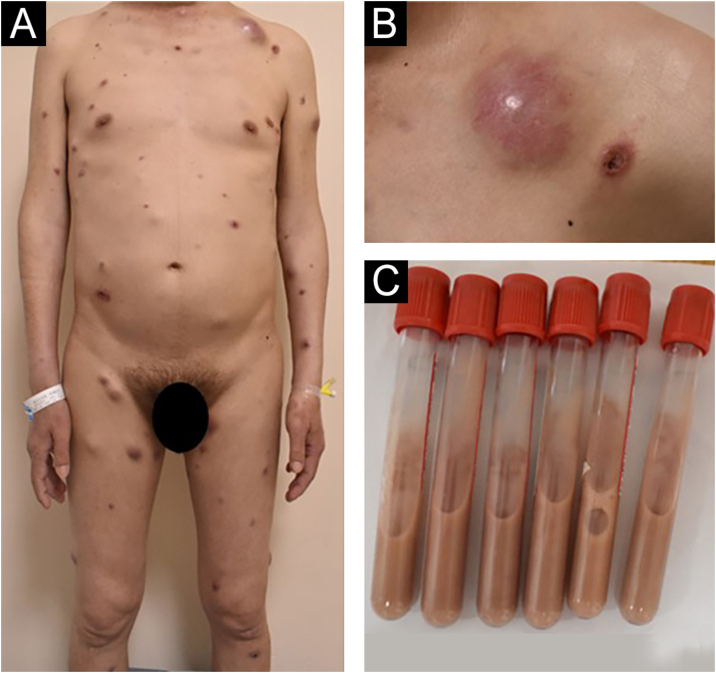
(A,B) Dermatological examination revealed erythematous and brownish papules, nodules and abscesses scattered throughout the body with varying sizes. Some lesions exhibited umbilicated central necrotic ulcerations and the abscesses were fluctuant and expressed yellow white purulent discharge. (C) The contents of the test tube are drawn from the abscess.

A 44-year-old male from Jiangxi Province, China, was admitted to the hospital for widespread papules and nodules lasting two months. Two months ago, several soybean-sized red papules and nodules appeared on the torso with no known triggers, with no significant pain or pruritus. After scratching, the lesion progressed into erosion and showed poor healing. The skin lesions gradually increased and enlarged, with an ulcer appearing in the center of the lesions. The patient claimed that he often walked in forests infested with bamboo rats before becoming ill. He had a 17-year history of syphilis, a 5-year history of genital herpes, and a history of soliciting a prostitute. He denied any history of drug use, homosexual activity, or blood transfusions. Vital signs on admission were stable. A physical examination revealed an emaciated appearance, hepatosplenomegaly, and bilateral lympadenopathies on the neck, axilla and groin with a normal neurological examination. Dermatological examination revealed erythematous and brownish papules, nodules and abscesses scattered throughout the body with varying sizes. Some lesions exhibited umbilicated central necrotic ulcerations and the abscesses were fluctuant and expressed yellow-white purulent discharge (Fig. 1).

Laboratory test results were as follows: white blood cell Count (WBC): 24.34 × 10^9^/L, hemoglobin (Hb) level: 62 g/dL, platelet count: 366 × 10^9^/L, absolute neutrophil count (ANC): 20.93 × 10^9^/L, albumin level: 18.4 g/L, C-reactive protein level: 187.41 mg/L, erythrocyte sedimentation rate (ESR): >140 mm/h, ferroprotein level: 763 ug/L. Detection of serum immune markers of the hepatitis B virus (5 items) indicated that hepatitis B surface antigen, e antibody, and core antibodies were positive. HIV serology was negative, with negative anti-interferon-γ autoantibodies. No connective tissue diseases or other immunodeficiency diseases were found. A high-resolution computed tomography (HRCT) of the cervicothoracic whole abdomen suggested multiple enlarged lymph nodes in the neck, mediastinum, bilateral axilla, hilar region, retroperitoneum, and bilateral groin with partial liquefaction and necrosis.

**Figure 2:**
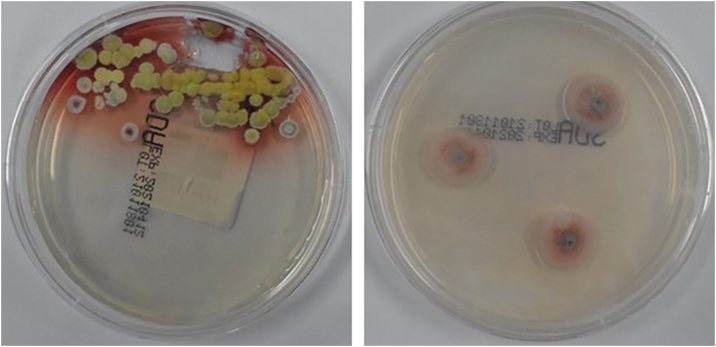
Colonies of *Talaromyces marneffei* were cultured from purulent secretions of skin lesions.

**Figure 3:**
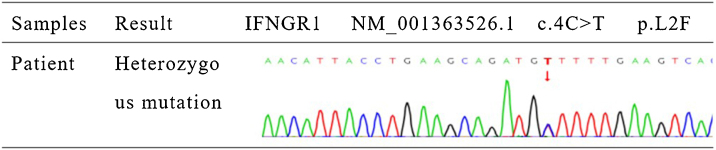
Sanger sequencing validation: *IFNGR1* gene mutation was detected in the patient (c.4C>T; p.L2F).

Subpleural abnormal density shadow in the lower lobe of both lungs, initial hypostatic effect. Cultures of purulent secretions from the rash revealed TM infection (Fig. 2). Galactomannan test, bone marrow puncture culture, blood culture, stool and urine culture, and bronchoalveolar lavage fluid culture showed no obvious abnormalities. In addition, lymph node biopsy showed reactive hyperplasia without evidence of fungal elements. With the consent of the patient, the deoxyribonucleic acid (DNA) samples were extracted from his peripheral blood. *IFNGR1* gene (NM_001363526.1) mutation was detected using whole exome sequencing (WES). The result was validated using Sanger sequencing for the detected mutation and the heterozygous mutation c.4C>T (p.L2F) in the identified *IFNGR1* gene (NM_001363526.1) (Fig. 3).

The patient was given intravenous voriconazole antifungal therapy according to the TM culture and drug-sensitivity test results. On the third day of administration, the patient’s body temperature and heart rate returned to normal, the lesions gradually healed, and the lymphadenopathy and hepatosplenomegaly improved. After seven days of voriconazole treatment, the patient’s Whole Exome Sequencing (WBC), C-reactive protein, and platelet counts were within normal limits. After one month of treatment, the skin mass was significantly reduced, and he is still being followed up.

## Discussion

In 1956, for the first time, at Institute Pasteur, TM was isolated from the liver of a bamboo rat that died of histoplasmosis.^3^ It is a temperature-dependent biphasic fungus distributed in Southeast Asia, northern India, and southern China.^4^ The yeast form is its main pathogenic form. The primary mode of infection of TM is by inhalation of spores. In the respiratory tract, the agent is converted to yeasts, spreading in the body by the blood system.^3^ In general, HIV infection is the single most important underlying condition leading to infection. However, in recent years, the number of patients with non-HIV and non-immunosuppressive TM infections has also increased. In this study, the patient was HIV-negative, lived in southern China all year round, and wandered among the forest infested with bamboo rats, which was at high risk of infection.

The pathogenesis of TM infection is not fully understood and mainly involves the mononuclear phagocyte system. Once the infection is established, the fungi spread by adhering to and colonizing host tissues, propagating, escaping, or destroying host defense systems, and damaging host tissues. The common clinical manifestations of TM infection include fever, weight loss, cough, anemia, generalized umbilicated papules, lymphadenopathy, and cavitary pneumonia.^5^ Patients with delayed diagnosis and antifungal treatment have a high mortality rate of about 75%, regardless of HIV infection.^6^ Chen Donghe et al.^7^ have reported cases of death caused by delayed diagnosis and treatment.

It has been documented that anti-interferon-γ autoantibodies are the most important factor causing severe TM infection in HIV-negative patients with adult-type acquired immunodeficiency.^8^ However, in this report, the anti-interferon-γ autoantibody test result of the patient was negative. Hence, the authors speculated that the primary causes of infection are two factors: the environmental factor mentioned above, and the other may be related to IFNGR gene mutation. *IFNGR1* is an important gene encoding Interferon-γ Receptor 1 (IFN-γR1), which is involved in the composition of a ligand-binding chain of the IFN-γ receptor. The *IFNGR1* mutation may change the structure of the IFN-γ receptor, thus affecting the binding and signal transduction of the IFN-γ receptor and interferon-γ, thus failing to effectively exert its biological effects. Interferon-γ is an important immunomodulatory cytokine produced by activated T-cells and Natural Killer (NK) cells (Th1 and CD8+ T-cells). It is a key mediator of the host immune response. After binding to the interferon-γ receptor, the signal transduced via the Janus Kinase-Signal Transducer and Activator of Transcription (JAK-STAT) pathway can activate or otherwise regulate the functional activity of mononuclear macrophages in other manners and induce the ability to kill various intracellular and extracellular microbial pathogens and tumor cells,^9^ which can promote the proliferation of T-lymphocytes and improve cellular immune function. It is hypothesized that *IFNGR1* gene mutation affects the binding and production of interferon-γ, causing immune deficiency or impaired immunity, thus increasing the risk of TM infection. In addition, the severity of the disease depends on the host’s immune status, including neutralizing anti-interferon-γ autoantibodies.^8^, ^10^ Six patients from the literature review and the one reported here had either a genetic mutation or positive anti-interferon-γ autoantibodies. After standardized anti-fungal therapy, the prognosis was good, with a low mortality rate. Only one patient with both the mutation and positive anti-interferon-γ autoantibodies died.^11^, ^12^, ^13^, ^14^, ^15^, ^16^ Therefore, it is speculated that gene mutation is a potential risk factor for TM infection, and the presence of anti-interferon-γ autoantibodies can aggravate disease symptoms. The co-existence of both may lead to poor prognosis and even increase the risk of death.

Amphotericin B remains the preferred drug in treating TM infection. A recent study has found that voriconazole has comparable efficacy to amphotericin.^6^ According to the results of the drug-sensitivity test of TM culture culture and considering the adverse effects of amphotericin B such as infusion reaction, liver and kidney function impairment, electrolyte disturbance, anemia, and patient tolerance etc,^17^ may aggravate the patient’s condition, the authors selected voriconazole for this patient. Voriconazole, administered in this patient, is the second-generation synthetic triazole broad-spectrum antifungal agent with a wide anti-fungal spectrum and strong anti-fungal activity. It is an effective and well-tolerated drug for treating penicilliosis,^18^, ^19^ with relatively few adverse events.

There have been no reported cases of non-AIDS, non-immunosuppressive, anti-interferon-γ autoantibodies negative, *IFNGR1* gene mutation of TM infection until now. The authors believe that genetic mutations are a potential risk factor for TM infection, and the presence of anti-interferon-γ autoantibodies can aggravate disease symptoms.

## Financial support

This work was supported by the Science and Technology Research Project of the Education Department of Jiangxi Province (nº GJJ200230) and the Health Commission Jiangxi Provincial of China (nº 202130178).

## Authors’ contributions

Shiyang Li: Data collection; drafting and editing of the manuscript.

Xianwei Cao: Data collection; drafting and editing of the manuscript.

Zhuxiu Guo: Data analysis; manuscript review.

Jian Wang: Data analysis; manuscript review.

Jianbo Tong: Concepts, drafting and editing of the manuscript; critical review of relevant intellectual content; approval of the final version of the manuscript.

Zhibin Zhang: Concepts, drafting and editing of the manuscript; critical review of relevant intellectual content; approval of the final version of the manuscript.

## Conflicts of interest

None declared.
